# Carotid artery injury from an airgun pellet: a case report and review of the literature

**DOI:** 10.1186/1746-160X-5-3

**Published:** 2009-01-17

**Authors:** Syed Abad, Ian DS McHenry, Lachlan M Carter, David A Mitchell

**Affiliations:** 1 Maxillofacial Surgery, Pinderfields General Hospital, Aberford Road, Wakefield, WF1 4DG, UK

## Abstract

Historically airguns were powerful weapons. Modern models, though less lethal, are still capable of inflicting serious or life threatening injuries. Current United Kingdom legislation fails to take into the account the capacity for airguns to maim and kill. We believe that airguns should be governed by the same law that applies to firearms. We present a case of a potentially fatal airgun injury to the neck. The airgun pellet caused a defect in the anterior wall of the external carotid artery, which required rapid access and surgical repair. We discuss the mechanism of airgun injury and review the literature in terms of investigation and management.

## Background

Historically airguns were powerful weapons. Modern models, though less lethal, are still capable of inflicting serious or life threatening injuries. Current United Kingdom legislation fails to take into the account the capacity for airguns to maim and kill. We believe that airguns should be governed by the same law that applies to firearms. We present a case of a potentially fatal airgun injury to the neck and review the literature.

## Case presentation

**Figure 1:**
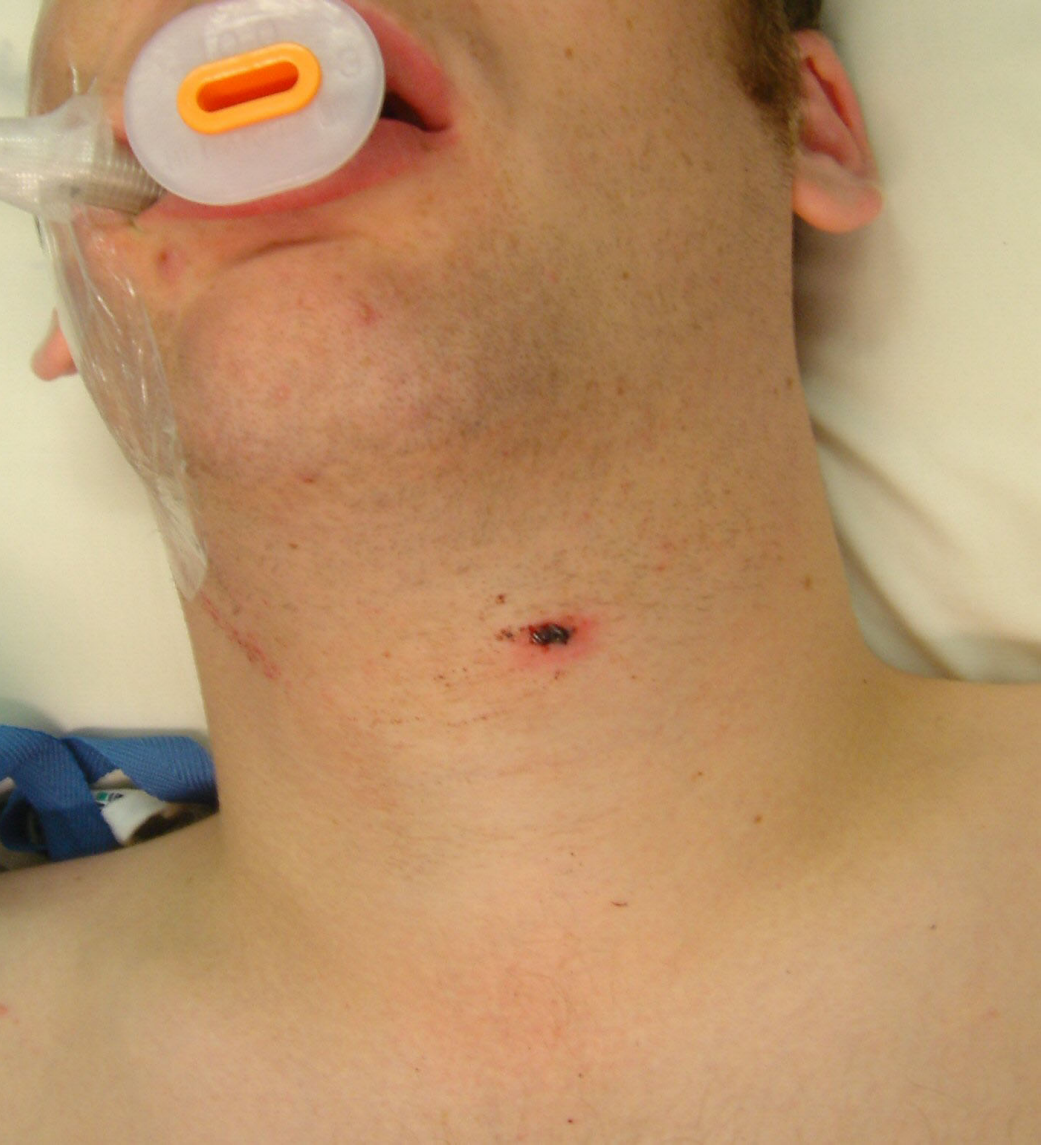
**Left neck entry wound**.

**Figure 2:**
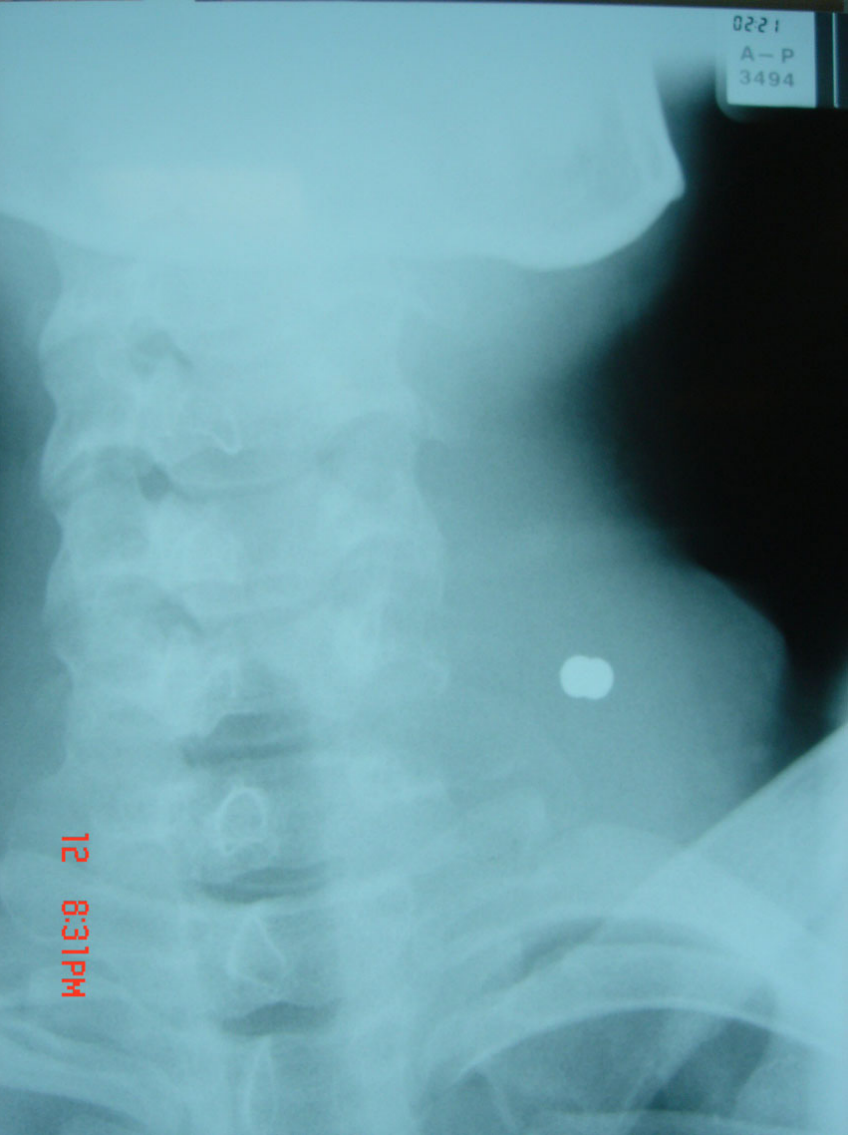
**Cervical spine radiograph showing pellet and haematoma**.

A 20-year old male presented to the Emergency Department having been accidentally shot in the neck, by a friend using a 0.22 calibre air rifle, at a distance of approximately three metres. He complained of neck stiffness and pain on swallowing. Examination revealed an entry wound 1 cm below the level of the thyroid notch to the left of the midline, figure 1. A tense, non-pulsatile haematoma was evident deep to the entry wound. No bleeding or surgical emphysema were present. His voice was hoarse, although flexible endoscopic examination showed no pharyngeal or laryngeal abnormality. Cervical spine radiographs demonstrated an airgun pellet antero-lateral to the transverse process of C6, with a surrounding 6 cm diameter radiopacity consistent with haematoma and no deviation of the trachea, figure 2.

**Figure 3:**
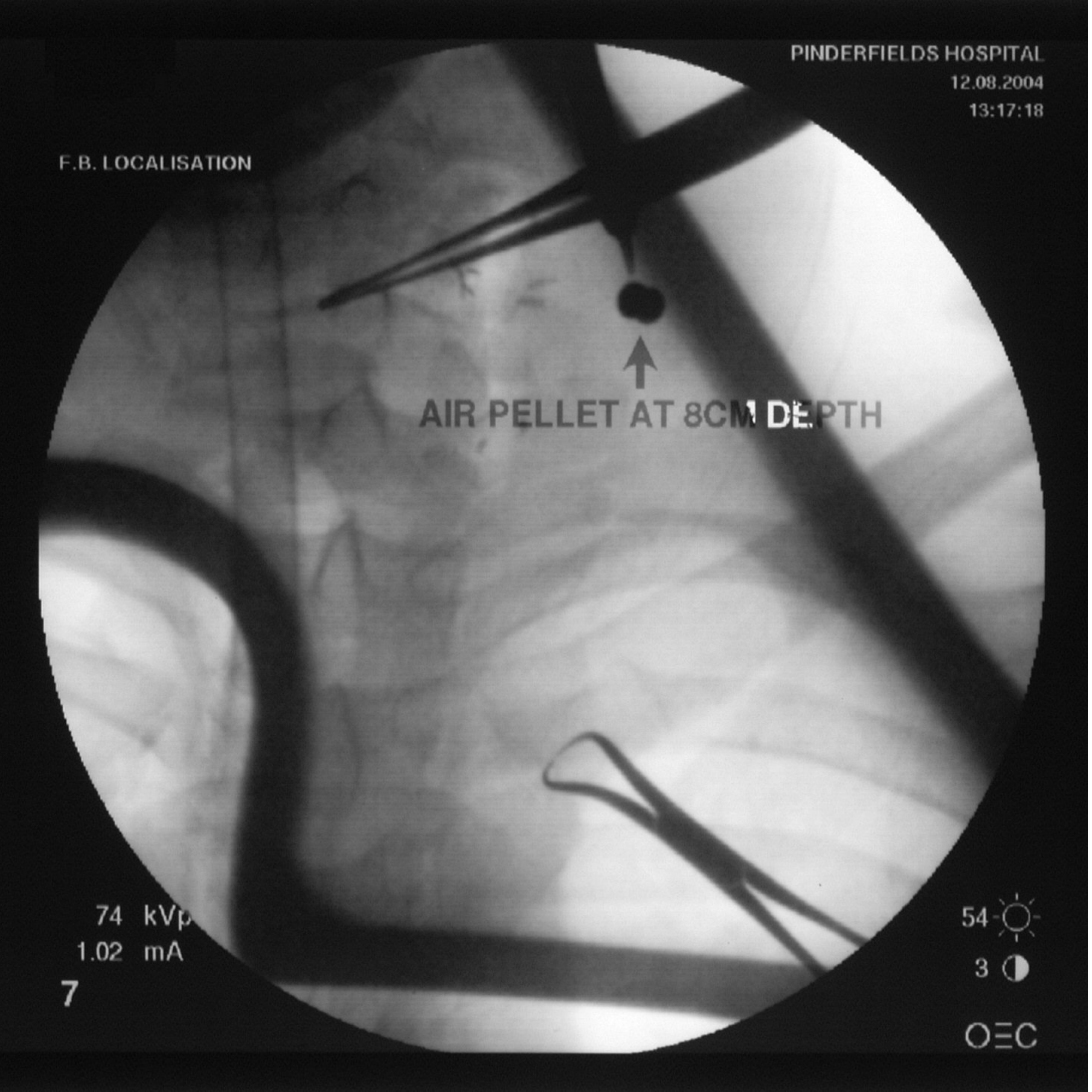
**Intra-operative image intensification views showing air gun pellet anterior to transverse process of C6 vertebra**.

Primary resuscitation was uneventful, and the patient was taken to theatre within four hours for exploration of the wound and evacuation of the haematoma. Upon evacuation of the haematoma extensive haemorrhage developed from a defect in the anterior wall of the external carotid artery, which required rapid access and surgical repair. The airgun pellet was localised using image intensification and successfully retrieved from the paraspinous muscles, figure 3.

**Figure 4:**
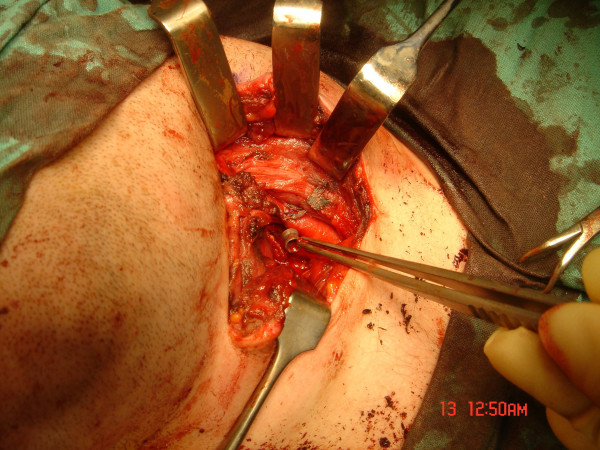
**Airgun pellet deep to external carotid artery**.

The pellet tract traversed the left lobe of the thyroid gland and the carotid sheath, impacting on the antero-medial aspect of the external carotid artery, just above the bifurcation, figure 4. It further traversed the paraspinous muscles and embedded immediately anterior to the transverse process of the sixth cervical vertebrae, at a depth of 8 cm from the skin entry wound.

Initial recovery was uneventful, however at follow-up it was clear that the patient had Horner's syndrome from injury to the left cervical sympathetic chain and carotid arteries.

## Discussion

Around 250BC, Ktesbias II of Egypt, first described the use of compressed air to propel a projectile. Modern airgun history began in the 15th century. These weapons were known as wind chambers and were designed using an air reservoir connected to a cannon barrel. These devices were capable of propelling a four pound lead ball over a distance of 500 yards, and able to penetrate 3 inch oak board. These weapons rivalled the power of gun powder based firearms of that time and came into use in the Napoleonic wars in the late 17^th ^and early 18^th ^centuries [1]. Various styles of airgun have existed since, some being able to kill a 500 lb stag elk at a range of 150 paces, or fire 15 – 20 rounds a minute. French and Austrian sniper divisions used these weapons, favouring their quieter nature.

With respect to modern airguns, the definition varies from country to country, but in the United Kingdom, air pistols generating more than 8.1J (6 foot pounds) or air rifles generating more than 16.2J (12 foot pounds) are considered firearms.

Therefore modern airguns are typically low powered due to safety concerns and legal restrictions. High powered designs utilizing various power sources (table 1) are becoming increasingly common and are still used for hunting. These rifles can propel a pellet beyond 1100 ft/s (330 m/s) – approximately the speed of sound, and produce a noise similar to a .22 calibre rim fire rifle. These higher powered air rifles have the advantage over powder firearm rifles, in that they do not require a licence, and can be effectively used for pest control [2].

**Table 1:** Airgun power sources

Power source	Mechanism	Muzzle velocity
Mechanical piston		
Spring piston	Spring loaded piston	1200 ft/s (370 m/s)
Gas ram	pressurized air/nitrogen built into the piston	
Pneumatic		
Multi/Single stroke	require pre-compression of air into the chamber using an on board lever	700 to 1000 ft/s
Pre-charged pneumatic (PCP)	Filled by decanting air from a reservoir to pre-compress air into the chamber	
CO_2_		
CO_2_	require a disposable pre-filled cylinder of CO_2_	400 to 600 ft/s

**Figure 5:**
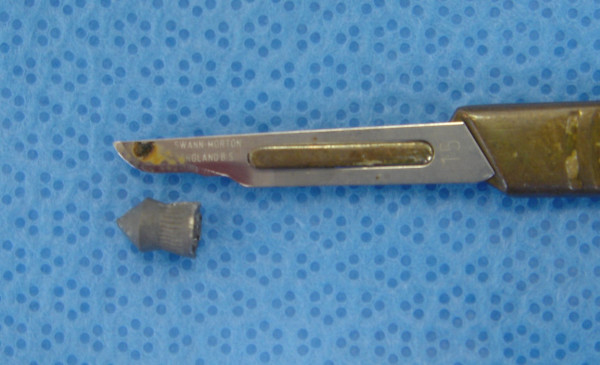
**Diabolo airgun pellet retrieved from the patient**.

The typical projectile used in rifled airguns is the lead diabolo pellet. This is a wasp waisted projectile open at the base, with a variety of head styles, figure 5. The flared tail of the pellet is designed to improve directional stability, as seen in a shuttle-cock.

Modern airgun pellets are capable of inflicting serious, if not life-threatening injuries. In the United Kingdom there is one death every year from airgun injury [3]. Despite recent advances in airgun technology, the legislation governing their use and sale remains largely unchanged. Airguns are capable of generating muzzle velocities of 350 ft/s or more. Furthermore a process known as 'dieseling' can make such weapons even more dangerous; petroleum oil is placed in the barrel and ignited by the heat generated from the passing pellet, resulting in an explosion which gives the pellet greater velocity and hence greater penetrating power. When a projectile strikes an object, energy from the projectile is transferred to its target. The kinetic energy of this projectile can be calculated by the following equation [4];

Kinetic energy = (mass × velocity × velocity)/2

High energy missiles can be defined as an object travelling at a speed in excess of 2000 ft/s. These high energy projectiles inflict damage on their targets by the processes of shock wave, temporary cavitation and permanent cavitation.

Low energy missile injuries occur at velocities below 1500 ft/s. These injuries, such as those produced by air rifles and guns, occur by a different process. Direct effects on tissues occur, such as laceration and crushing within the missile tract, rather than the effects of temporary cavitation.

The critical velocity required for penetration of human skin by an air rifle pellet is around 125–230 ft/s (38–70 m/s), which is within the muzzle velocities of many air rifles available for sale in the UK [1]. Muzzle velocity alone is not the only factor determining the damage that can be inflicted by an air rifle pellet. The pellet can rapidly loose velocity over distance and thus the pellet velocity at the target is more relevant in terms of tissue damage. Muzzle velocity does provide a useful scale for comparison of the power of air weapons.

In the U.K., recent changes to the law fall short of the restrictions needed to protect the public from the dangers of such devices. Any person aged 17 years or over can carry an airgun in a public place. Children of 14 years and over can fire an airgun unsupervised on a private land, whereas children under the age of 14 years can fire airguns only when supervised by an adult over 21 years [5].

Review of the literature has revealed an alarming trend in increasing incidence and severity of airgun pellet injuries. Most airgun pellet injuries occur in children and adolescents. The most common site is the head and neck region. The airway and neurovascular structures make the neck a potentially life threatening site of injury. Holland et al reported three cases of penetrating airgun injuries to the neck [5]; two had the pellet removed and one had conservative management. David [6] also published a case involving penetrating injuries to the neck in a 19-year old male, in whom the pellet was successfully removed from the posterior oesophageal wall. In our case the airgun pellet penetrated the robust structure of the carotid sheath producing an arterial bleed which was tamponaded by the surrounding tissue. This bleed, if not controlled, may have led to airway compression and death.

There are numerous accounts of airgun and ball-bearing injuries to the cranium and orbital tissues. Bruce-Chwatt et al [7] illustrated a case of Horner's syndrome due to an airgun pellet injury to the neck which subsequently resolved. Horner's syndrome occurred in our case with damage to the left carotid arteries and the cervical sympathetic chain as a possible causative mechanism. Unfortunately the patient has not attended for long-term follow up.

Plain radiographs are important in evaluation of a wound when an airgun pellet injury is suspected. Patients may be unaware of being shot. In addition the entry wound is often very small, thus serious injuries may be trivialised or missed completely. Image intensification was essential in retrieval of the pellet in our case. Airgun pellets can also be located by ultrasound guided techniques. This can minimize the need for blind exploration of wound tracts and thus limit complications such as swelling and haematoma formation by facilitating a smaller surgical wound [8].

Van As et al [9] advocated the use of selective angiography in management of gunshot wounds to the neck, together with careful clinical examination. Vascular imaging may have been useful in our case as identification of the bleeding source may have led to a wider surgical approach to the haematoma allowing control of the carotid artery more distant from the bleeding site. A wider, more open approach may have limited the intra-operative haemorrhage.

There is debate as to whether surgical exploration and retrieval of airgun pellets is necessary, particularly where the risks are deemed far greater than to simply leave the pellet in situ [9,10]. In our case, operative intervention was deemed necessary for evacuation of the haematoma and control of haemorrhage. Fortunately retrieval of the pellet was also possible.

High velocity missile injuries of the maxillofacial region can be entirely different from those caused by low velocity projectiles. High velocity wounds are dangerous because they carry the risk of airway obstruction due to direct or indirect laryngeal obstruction, particularly when wounds are closed [11]. High powered airguns augmented with the dieseling process may be able to produce muzzle velocities sufficient enough to be considered high energy and thus at short range may cause shock wave, temporary and permanent cavitation. However, high velocity projectiles can cause low energy wounds, as the energy transferred dictates the type of wound formed, not the initial velocity of the missile. The rate of energy transfer may also vary along the wound tract [12].

Low velocity, low-calibre injuries in the maxillofacial region are rarely fatal due to haemorrhage or airway obstruction. Most vascular injuries can be treated by observation, but angiography is a necessity if a missile enters the base of skull or neck. If a projectile cannot be found in the area of the missile tract, it may have embolised within a vessel, and transported to a distant site [13].

## Conclusion

In summary, the type of wound formed, rather than the weapon responsible, dictates the treatment required. Although airguns and air rifles are not considered serious weapons, they can produce injuries with serious morbidity, or even mortality, particularly in young adults [2]. Angiography should be considered, particularly in wounds involving the neck or base of skull. Other non invasive techniques, such as ultrasound, can be a useful adjunct for locating projectiles. As airgun pellets and firearm rounds are not sterilized by firing, these foreign bodies can introduce infection, even if temporary cavitation is not formed.

Airguns are capable of inflicting serious injury as demonstrated by our case. The incidence and severity of such injury is increasing. We believe that airguns should be governed by the same laws that apply to firearms. Doctors and emergency personnel need to be aware that airgun pellet injuries can be fatal and should not be trivialised. Careful, thorough history and examination and appropriate imaging are imperative in the management of such injuries.

Many authors have rallied for a change in legislation to take into account the severity of airgun pellet injuries. To date this has not yet materialised.

## Competing interests

The authors have no financial and personal relationships with other people, or organisations, that could inappropriately influence (bias) their work, all within 3 years of beginning the work submitted.

## Authors' contributions

SA and LMC prepared the case report. SA, IDSM and LMC prepared the discussion. LMC and DAM edited the discussion and prepared the final draft of the paper. DAM conceived the paper and prepared the figures. All authors read and approved the final draft of the manuscript.

## Consent

Unfortunately, the patient could not be traced to obtain written informed consent. We believe that this case report contains a worthwhile clinical lesson which could not be made as effectively in any other way. We expect that a reasonable person would not object to the publication since every effort has been made so that the patient remains anonymous.
